# Gene coexpression network analysis for family studies based on a meta-analytic approach

**DOI:** 10.1186/s12919-016-0016-y

**Published:** 2016-10-18

**Authors:** Renaud Tissier, Hae-Won Uh, Erik van den Akker, Brunilda Balliu, Spyridoula Tsonaka, Jeanine Houwing-Duistermaat

**Affiliations:** 1 Department of Medical Statistics and Bioinformatics, Leiden University Medical Center, PO Box 9600, 2300 RC Leiden, The Netherlands; 2 Molecular epidemiology, Leiden University Medical Centre, Leiden, The Netherlands; 3 Pattern Recognition & Bioinformatics, Delft University of Technology, Leiden, The Netherlands; 4 Department of Statistics, University of Leeds, Leeds, UK

## Abstract

For a better understanding of the biological mechanisms involved in complex traits or diseases, networks are often useful tools in genetic studies: coexpression networks based on pairwise correlations between genes are commonly used. In case of a family-based design, it can be problematic when there is a large between-family variation in expression levels. We propose here a gene coexpression network analysis for family studies. We build a coexpression network for each family and then combine the results. We applied our approach to data provided for analysis in the Genetic Analysis Workshop 19 and compared it to 2 naïve approaches—ignoring correlations among the expressions and decorrelating the gene expression by using the residuals of a mixed model—and a single-probe analysis. Our approach seemed to better deal with heterogeneity with regard to the naïve approaches. The naïve approaches did not provide any significant results, while our approach detected genes via indirect effects. It also detected more genes than the single-probe analysis.

## Background

Weighted gene coexpression network is a widely used method for studying biological networks based on pairwise correlations. This method provides more insight in the underlying biological mechanisms and offers a tool for dimension reduction by summarizing identified modules (clusters) of genes [1, 2]. How to perform such an analysis for family data is an open question. For family data Kraft et al. [3] noted that testing association between expression levels and traits without taking into account the family structure can lead to spurious results, especially when the number of families is small and in the presence of large between-family variation. In this paper, we propose a novel strategy for network analyses in a small set of relatively large families.

For this family-based approach, we first construct family-specific coexpression networks and test for association between the modules and the traits of interest. A common set of genes for all families was obtained by using the intersection and the union of family-specific modules. We compare this family-based approach with 2 naïve approaches: namely, one using the gene expression of the families directly (ignoring correlation) and one that first decorrelates the gene expressions and then applies the standard approach. We also compare our results with single-probe analyses.

## Methods

### Study sample

The gene expression data set is composed of 647 individuals from 17 large families. These samples are from the data set described in Almasy et al. [4]. Here, we focus on the largest 5 families: namely families 2, 5, 6, 8, and 10 with 65, 55, 45, 62, and 49 family members, respectively. The total number of individuals is 276 and the total number of probes from which gene expression is available is 20,364. We used the simulated quantitative phenotypes systolic blood pressure (SBP) and the phenotype Q1 at time point 1 as outcome variables. The simulation model of SBP comprises 15 genes and that of Q1 does not contain any of these genes. SBP, Q1, and all probes were corrected for age and sex by regressing out covariates and using residuals.

To decorrelate the gene expressions, we fitted for each probe a linear mixed model: *X*_*ij*_ = *μ* + *u*_*ij*_ + *v*_*i*_ + ε_*ij*_, with *X*_*ij*_ the value of the probe for the individual *j* in family *i*, *u*_*ij*_ a normally distributed random genetic effect: *u*_*ij*_ ~ N(0, S) where S = 2 * K * *s*_*g*_ with K kinship matrix and *s*_*g*_ genetic variance, *v*_*i*_ a normally distributed random effect representing shared environmental effects, and ε_*ij*_ a normally distributed residual. To obtain the residuals *X*_ij_^*^ of this model, we used the function *lmekin,* which fits linear mixed models with specific structure of the variance-covariance matrix from the package *coxme* [5] in R.

### Single-probe analysis

For the single-probe analysis the following mixed model was used:

1 $$ {Y}_{ij} = \mu +{u}_{ij}+{v}_i+\beta {X}_{ij}+{\upvarepsilon}_{ij} $$

with *Y*_*ij*_ the value of SBP or Q1 and *X*_*ij*_ the value of the probe for individual *j* of family *i*. The random effects *u*_*ij*_, *v*_*i*_ and ε_*ij*_ are the genetic effect, the shared environmental effect and residuals respectively. The parameter β represents the effect of the probe on the outcome variable.

### Network constructions

Coexpression networks were built on the data set without correction for family structure based on *X*_*ij*_ (naïve approach), the data set adjusted for family structure based on *X*_ij_^*^ (naïve decorrelated approach), and on the data sets from the 5 families separately.

We used signed coexpression networks. The adjacency matrix *A* = [*a*_*lk*_] of each network was computed as follows: *a*_*lk*_ = |0.5 + 0.5 × *cor*(*x*_*l*_, *x*_*k*_)|^*γ*^, with *cor*(*x*_*l*_, *x*_*k*_) the correlation between *x*_*l*_ the values vector of probe *l* and *x*_*k*_ the values vector of probe *k*. The parameter *γ* is acting as a soft threshold in the adjacency matrix, when we increase the value *γ* the coefficient of the adjacency matrix will tend toward zero except for values really close to 1. We used the biweight midcorrelation based on the median, which is more robust than the Pearson correlation. The co-expression networks were constructed with the R package WGCNA (weighted gene correlation network analysis) [6]. For each obtained module, the first principal component (eigengene) was computed.

### Phenotype analysis

From all modules and all families, the following models were fitted:

2 $$ {Y}_j = \mu +{u}_j+\beta eigengen{e}_j^k+{\upvarepsilon}_j $$

where *Y*_*j*_ is the outcome, *u*_*j*_ the random genetic effect, and eigengene_j_^k^ the value of the eigengene of module *k* of family member *j.* Let E_*F*2_^*M*^ to E_*F*10_^*M*^ be the most significant eigenvalues of the family specific networks (N_F2_ to N_F10_) and let E_*F*_^*M*^ be the most significant eigenvalue of these 5 eigenvalues and M_*F*_^*M*^ be the corresponding module. Identify the modules of the family-specific networks, which have the highest overlap with M_*F*_^*M*^ (denoted as M_*F*2_^*O*^ to M_*F*10_^*O*^). Next, 2 common sets of genes for all families were obtained by taking the intersection (M_F_ = M_*F*2_^*O*^ ∩ M_*F*5_^*O*^ ∩ M_*F*6_^*O*^ ∩ M_*F*8_^*O*^ ∩ M_*F*10_^*O*^) and the union (M_F_ = M_*F*2_^*O*^ ∪ M_*F*5_^*O*^ ∪ M_*F*6_^*O*^ ∪ M_*F*8_^*O*^ ∪ M_*F*10_^*O*^) of the family specific modules. The first principal components of the 2 common sets were computed. The principal component that explained most of the variance of the corresponding set of genes was used as the eigengene E_F_ of the family-based approach.

The eigengenes of the naïve approach (E_N_), the naïve approach after decorrelation (E_ND_), and the family-based approach (E_F_) are tested for association with the 2 phenotypes SBP and Q1. Here, the following mixed model was used:

3 $$ {Y}_{ij} = \mu +{u}_{ij}+{v}_i+\beta eigengen{e}_{ij}^k+{\upvarepsilon}_{ij} $$

with *Y*_*ij*_ the phenotype value for individual *j* of family *i* and *eigengene*_*ij*_^*k*^ the value of eigengene of module *k* of individual *j* of family *i*. And *u*_*ij*_, *v*_*i*_ and ε_*ij*_ are the genetic effect, the shared environmental effect and residuals, respectively. The parameter *β* represents the effect of the eigengene *k* on the outcome variable.

Finally, because spurious associations are especially expected in the presence of large between-family heterogeneity [1], we also performed a network analysis using the subset of 25 % most heritable probes when performing the network analysis (*n* = 4911 probes with heritability between 0.33 and 0.88).

To test for significance we used a nominal alpha level of 0.05 and the Bonferroni correction was applied to take into account multiple testing.

## Results

### Results obtained with all probes

For per family analysis, the module that showed the highest correlation with the SBP was the magenta module obtained in family 8 (M_*F*8_^*M*^) (β = 2.52, *p* = 0.0011). M_*F*8_^*M*^ comprises 710 genes. Table 1 gives for each family the number of genes of the module with the highest overlap. The intersection and the union of these 5 family modules, comprises 62 and 1746 probes, respectively. The first principal component (eigengene) of the probes in the intersection set explained more than 50 % of the variance for each family, while for the union set the eigengene explained only between 23 % and 31 % of the variance of the expression levels. Therefore the eigengene of the intersection set was used as summary for the family approach (E_F_). In Table 2, for each family the effect of E_Fi_ on SBP (β of model (2)) is given. For families 2 and 8, the eigengenes (E_F2_ and E_F8_) were significantly associated with SBP.

**Table 1:** Module size of M_*F*2_^*O*^ to M_*F*10_^*O*^ and overlap size with M_*F*_^*M*^ in the all-probes analysis

	M_*F*2_^*O*^	M_*F*5_^*O*^	M_*F*6_^*O*^	M_*F*8_^*O*^	M_*F*10_^*O*^
Module size	446	694	499	710	446
Size of the overlap with M_*F*_^*M*^	187	308	240	710	372

**Table 2:** Parameter estimates of the association between eigengenes and Q1 and SBP

	All-probes		25 % Most heritable probes	
	SBP	Q1	SBP	Q1
E_F2_	−0.57(0.2) (0.02)^a^	9.90(4.3) (0.02)	0.27(0.1) (0.07)	−1.62(1.0) (0.11)
E_F5_	0.34(0.2) (0.21)	14.0(4.7) (3.3e-3)	0.18(0.2) (0.41)	−2.13(1.3) (0.11)
E_F6_	0.08(0.3) (0.78)	8.90(3.7) (0.02)	0.66(0.3) (0.01)^a^	2.49(0.9) (9.6e-3)
E_F8_	−0.62(0.3) (0.04)^a^	10.47(4.2) (0.01)	0.07(0.2) (0.68)	2.47(1.0) (0.02)
E_F10_	0.14(0.3) (0.67)	7.55(4.5) (0.09)	0.02(0.2) (0.91)	−2.22(1.2) (0.06)
E_F_	−0.13(0.2) (0.49)	-	0.21(0.09) (0.02)^a^	-
E_N_	−3.21(1.3) (0.01)	2.75(0.7) (5.6e-4)^a^	1.93(0.8) (0.01)	−0.96(0.5) (0.06)
E_ND_	−3.03(1.1) (6.1e-3)	−1.41(0.4) (9.4e-4)^a^	1.94(0.7) (5e-3)^a^	−0.41(0.2) (0.06)

In parentheses are standard errors and *p* values, respectively. For E_F2_ to E_F10_ model (2) was used; for E_F_, E_N_ and E_ND_ model (3) was used. For Q1 the association results for E_*F*2_^*M*^ to E_*F*10_^*M*^ are presented

^a^ Denotes significant test after multiple testing corrections

When analyzing all families together, none of the approaches provided significant results. The joint analysis of the families using E_F_ as eigengene in model (3) did not provide a significant association SBP (β = −0.13, *p* = 0.49). For the naïve approach, the eigengene of the module magenta (E_N_) had the smallest *p* value (β = −3.21, *p* = 0.01). For the naïve approach using the decorrelated data set, the eigengene of the module grey60 (E_ND_) had the smallest *p* value (β = −3.03, *p* = 0.0061). After multiple-testing correction (between 43 and 50 modules in each network) none of the results were significant. Finally the single-probe analysis preformed in the 5 families by using model (1) provided 1 significantly associated probe with SBP (*CRIP2*; β = −13.68, *p* = 1.7e-06).

The intersection module of the family based approach did not contain any of the 15 genes used for the simulation. Also, the identified gene of the single-probe analysis is not among these 15 genes. We hypothesized that correlation might exist between E_F2_, E_F8_, and the gene expression of *CRIP2* on one hand and the set of 15 genes on the other hand. Indeed, E_F2_ showed significant correlation with *PSMD5* (*p* = 0.004) and *GTF2IRD1* (*p* = 0.007), and E_F8_ showed significant correlation with *ZNF443* (*p* = 5e-05), *PSMD5* (*p* = 3e-05), and *ABTB1* (*p* = 6e-05). When the presence of these 15 genes in the modules was investigated, it appeared that they were in different modules (Table 3). The gene *CRIP2*, which was significant in the single-probe analysis, showed significant correlation with the gene *KRTAP11-1* (*p* = 3.1e-03).

**Table 3:**
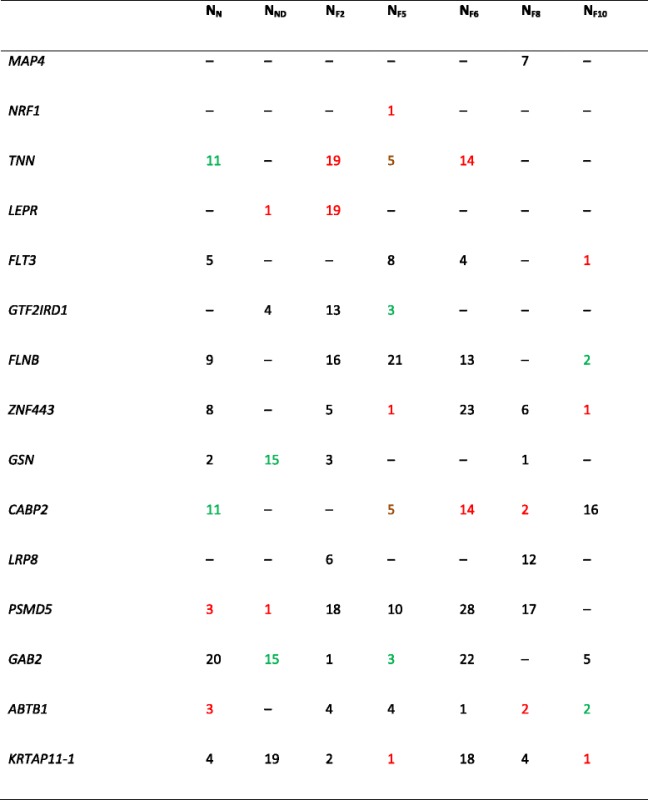
List of the top genes involved in the simulation model and their module color in each network

–, Denotes the grey module in which all nonclustered genes are combined. The different colors represent genes in the same module for a specific network

### Analysis of Q1

The results of the analysis of Q1 are also given in Table 2. For the family approach, none of the modules obtained in family-specific network analysis was significantly associated with Q1 and no common set could be defined. In Table 2, the estimates of strongest associated modules E_*F*_^*M*^ for each family are given. For the naïve approach, the module red (E_N_) (β = 2.75, *p* = 0.00056) was significant and for the naïve approach using the decorrelated data the module green (E_ND_) (β = −1.41, *p* = 0.00094) was significantly associated with Q1.

### Results obtained with the 25 % most heritable probes

For the naïve and the family approaches, the results of the network-based analyses using only the gene expressions of the 25 % most heritable probes (*n* = 4911 probes with heritability between 0.33 and 0.88) are also given in Table 2. None of the 15 genes used in the simulation model for SBP was among this set of most heritable probes. For Family 6 the E_F6_ was significantly associated with SBP (*p* = 0.01). The association of E_F_ in the 5 families with SBP was also significant (*p* = 0.02). For Q1, none of the approaches provided significant results. With regard to the single-probe analysis, no probe other than *CRIP2* was significantly associated.

## Discussion

In this paper, we have proposed a novel strategy to perform a coexpression network analysis with family data: building a network for each of the large pedigrees and defining a common module by taking the intersection of family-specific modules. We compared our family-based approach with 2 naïve network approaches and a single-probe analysis. All analyses were performed in a small set of 5 relatively large families. None of the 15 genes in the simulation model was identified in this small data set. However, the family-based approach identified significant associations between the eigengene and SBP in 2 families. This eigengene was significantly correlated with 4 of the 15 genes. When analyzing all families jointly the family eigengene was not significant. Also the naïve network approaches did not provide any significant result. The single-probe analysis provided 1 significant gene, which was correlated with 1 of the 15 genes. To study the performance of the methods with regard to false-positive findings, we also analyzed the trait Q1 for which no gene expressions were included in the simulation model. The family approach did not provide a significant finding, whereas both naïve approaches identified a significant module for Q1. The result in the naïve approach based on gene expression (*X*_*ij*_) is in line with the findings of Kraft et al. [3]. We did not expect to have a false-positive finding when using the decorrelated data (*X*_ij_^*^) as input for our network analysis. Possible explanations for this finding are the fact that the correlation based on the kinship coefficient might not be appropriate for gene expressions, and randomness. In addition to the set of all probes, networks were also built using only the 25 % most heritable probes. Especially for these variables that show large between-family variation spurious associations might occur when the family structure is not taken into account. This was not confirmed in our analysis. More research is needed to study the sensitivity of the methods for between-family variation.

We did not know the answers when we developed the family-based approach and analyzed the data. The simulation model used to create the data sets may not be well suited to pick up the 15 genes directly by network analysis. The 15 genes present in the model were in different pathways: they were not correlated. Moreover our approach was able to identify indirect effects: that is, the eigengenes were correlated with the 15 genes. Thus the significant association of the family-based network approach represented the largest number of genes from the simulation model. We expect that especially in the presence of large between-family variation our approach would perform best. A thorough simulation study is required to investigate the performance of our method further.

Network analysis provides a tool to reduce the number of tests by first summarizing the data in sets of genes with correlated gene expressions and summarizing the gene set by the first principle component. Another obvious reduction step is to only consider the heritable probes for the analysis. It appeared that by using the heritable probes the results across the families were less heterogeneous. The family approach, as well as the naïve approach using decorrelated data, provided significant results for SBP.

## Conclusions

In this paper we combined the family-specific modules by taking the intersection of the modules which showed most overlap. This approach worked well for the relatively small set of 5 families. When we applied our method to the 6 largest families, similar results were obtained (data not shown). However, intersection might not be the most appropriate approach to combine modules across families, because the intersection set becomes too small. Alternative approaches have to be developed. For example, LASSO (least absolute shrinkage and selection operator)-type methods can be used to select probes from the union sets. Development of a method for constructing a common set from the family specific modules is a topic for future research.

Finally more research is needed to evaluate the performance of our method with regard to false-positive and false-negative findings in relationship to heterogeneity, family size, the number of families, and the heritability of gene expressions.
